# A story of resilience: Arctic diatom *Chaetoceros gelidus* exhibited high physiological plasticity to changing CO_2_ and light levels

**DOI:** 10.3389/fpls.2022.1028544

**Published:** 2022-11-11

**Authors:** Haimanti Biswas

**Affiliations:** ^1^ National Institute of Oceanography - CSIR (Council of Scientific and Industrial Research), Biological Oceanography Division, Goa, India; ^2^ Polar Biological Oceanography, Alfred Wegener Institute of Polar and Marine Research, Bremerhaven, Germany

**Keywords:** Arctic ocean, climate change, phytoplankton, diatoms, ocean acidification, multiple stressors

## Abstract

Arctic phytoplankton are experiencing multifaceted stresses due to climate warming, ocean acidification, retreating sea ice, and associated changes in light availability, and that may have large ecological consequences. Multiple stressor studies on Arctic phytoplankton, particularly on the bloom-forming species, may help understand their fitness in response to future climate change, however, such studies are scarce. In the present study, a laboratory experiment was conducted on the bloom-forming Arctic diatom *Chaetoceros gelidus* (earlier *C. socialis*) under variable CO_2_ (240 and 900 µatm) and light (50 and 100 µmol photons m^-2^ s^-1^) levels. The growth response was documented using the pre-acclimatized culture at 2°C in a closed batch system over 12 days until the dissolved inorganic nitrogen was depleted. Particulate organic carbon and nitrogen (POC and PON), pigments, cell density, and the maximum quantum yield of photosystem II (Fv/Fm) were measured on day 4 (D_4_), 6 (D_6_), 10 (D_10_), and 12 (D_12_). The overall growth response suggested that *C. gelidus* maintained a steady-state carboxylation rate with subsequent conversion to macromolecules as reflected in the per-cell POC contents under variable CO_2_ and light levels. A substantial amount of POC buildup at the low CO_2_ level (comparable to the high CO_2_ treatment) indicated the possibility of existing carbon dioxide concentration mechanisms (CCMs) that needs further investigation. Pigment signatures revealed a high level of adaptability to variable irradiance in this species without any major CO_2_ effect. PON contents per cell increased initially but decreased irrespective of CO_2_ levels when nitrogen was limited (D_6_ onward) possibly to recycle intracellular nitrogen resources resulting in enhanced C: N ratios. On D_12_ the decreased dissolved organic nitrogen levels could be attributed to consumption under nitrogen starvation. Such physiological plasticity could make *C. gelidus* “ecologically resilient” in the future Arctic.

## Introduction

The Arctic Ocean is one of the most vulnerable marine ecosystems to climate change (Smetacek and Nicol, 2005) mainly due to rising temperature (Bintanja, 2018), retreating sea ice, and the associated increase in light penetration (Arrigo et al., 2008; Nicolaus et al., 2012), and the accelerated rate of ocean acidification (OA) (Bellerby, 2017; Qi et al., 2017). Such changing hydrography and physicochemical conditions in the Arctic are affecting phytoplankton dynamics (Ardyna and Arrigo, 2020) and are important to understand. More specifically, light and CO_2_ levels may have immediate consequences on the Arctic phytoplankton physiology and carbon cycling. Light is one of the most crucial factors that control phytoplankton bloom in the Arctic waters (Arrigo et al., 2008; Arrigo et al., 2012; Ardyna et al., 2020). An increase in light stress may impede a steady photosynthetic performance (Nixon et al., 2010; Li et al., 2016). Additionally, due to increased light penetration associated with retreating sea ice and stratification, the chances of photoinhibition may enhance (Leu et al., 2016) leading to reduced photosynthetic rates. Diatoms are the major players in phytoplankton bloom development in the Arctic waters (Lovejoy et al., 2002), and changing irradiance levels directly modulate their community dynamics. Croteau et al. (2022) observed that the spring bloom succession is controlled by the shift in growth light optima among the bloom-forming Arctic diatom species. However, the experimental studies revealed that Arctic diatoms possess high photophysiological plasticity to adjust to such fluctuating light levels (Lacour et al., 2018; Lacour et al., 2019; Hoppe, 2021). However, other stress factors like increasing CO_2_ levels in combination with variable irradiance may affect the carbon capture potential of the bloom-forming diatoms in the Arctic, and such studies on multiple stressors are scarce from this region.

Persistent low temperatures in the Arctic water are responsible for the quick dissolution of CO_2_ and accelerate the rate of ocean acidification compared to other oceanic areas (Barnes and Tarling, 2017). In addition to this, the influx of Pacific low pH waters, and the freshwater input from ice melts are causing a quick rate (with spatial variability) of decreasing surface seawater pH and increasing CO_2_ levels (Arctic Monitoring and Assessment Programme: AMAP Assessment, 2018). But how Arctic phytoplankton would respond to such changing pH/CO_2_ levels is still not clearly understood and is mostly dependent on their carbon metabolism patterns that are known to be regulated in response to changing carbonate chemistry (Hopkinson et al., 2011; Young and Morel, 2015). Among the dissolved inorganic carbon (DIC) species, the dissolved CO_2_ level is usually<1% in the surface ocean whereas, bicarbonate ions (HCO_3_^-^) contribute to 90% of the oceanic DIC pool. Importantly, for the carboxylating enzyme Rubisco, CO_2_ is the sole substrate and its concentration in the surface ocean (10 -12 µmol kg^-1^) is significantly lower than its half-saturation constant (40 µmol kg^-1^; Badger et al., 1998). To achieve steady state carbon fixation rates, a majority of marine phytoplankton run carbon dioxide concentration mechanisms (CCMs) to maintain a higher ratio of CO_2_:O_2_ levels in the proximity of Rubisco. Such CCMs can be biophysical or biochemical, although the former mechanism was found to prevail in the natural marine diatom population (Pierella Karlusich et al., 2021). In this process, bicarbonate ions are pumped actively inside the cell (Morel et al., 1994; Badger, 2003) with consequent conversion to CO_2_ by a metalloenzyme carbonic anhydrase (CA) (Hopkinson et al., 2011; Reinfelder, 2011). The ongoing increase in surface seawater *p*CO_2_ is expected to facilitate higher diffusive CO_2_ influx inside the cell and decrease the diffusive loss of CO_2_ from the cell (Reinfelder, 2011). Subsequently, the energy and resource allocation to run CCMs can be downregulated providing energetic savings (Hopkinson et al., 2011) and growth enhancement. Nonetheless, decreasing cellular pH due to enhanced CO_2_ influx may also increase dark respiration rates (Wu et al., 2010) resulting in reduced growth. But how Arctic phytoplankton respond to such changing pH/CO_2_ levels remains inconclusive.

Previous experimental studies revealed large species-specific variability in their responses to elevated CO_2_ levels (Wolf et al., 2017; Kvernvik et al., 2020; Wolf et al., 2022). Some studies documented higher resilience of Arctic diatoms to ocean acidification and increasing irradiance levels (Hoppe et al., 2017; Hoppe et al., 2018a; Hoppe et al., 2018b) compared to the Antarctic species (Hoppe et al., 2015; Heiden et al., 2016). Increasing temperature alone (Coello-Camba et al., 2015) and in combination with ocean acidification were seen to impact the Arctic phytoplankton negatively (Coello-Camba et al., 2014). Open water diatoms showed higher plasticity under elevated CO_2_ and light levels compared to a sea-ice diatom (Kvernvik et al., 2020; Wolf et al., 2022). And such variable responses make it difficult to predict whether the cumulative impact of increasing CO_2_ and fluctuating irradiance on Arctic diatom physiology would be antagonistic or synergistic. Hence, the responses of bloom-forming diatoms, which contribute substantially to carbon capture, to changing irradiance as well as to higher CO_2_ levels are important to address.

The centric diatom *Chaetoceros gelidus* (earlier known as *Chaetoceros socialis*) (Chamnansinp et al., 2013) is a significant player in bloom development in the Arctic water (Booth et al., 2002; Balzano et al., 2017) and usually dominates the deep chlorophyll maxima (Martin et al., 2010). Previous experimental studies showed that *C. gelidus* was capable of adjusting under changing light and CO_2_ supply without compromising the growth. The competitiveness of this species was found to be quite high in response to variable light as well as nitrogen sources (Schiffrine et al., 2020). Hoppe et al. (2018a) conducted an onboard incubation experiment on the natural phytoplankton assemblage from the Baffin Bay in summer by mimicking the upwelling event when the phytoplankton from the subsurface (low light) are brought up to the surface (high light). The community was dominated by *C. gelidus* and showed no significant response to either variable CO_2_ (380 and 1000 µatm) or light (15% and 35% incident solar light) levels. In another experiment conducted in the same location (Hussherr et al., 2017) the dominance of this species was noticed in all treatments with a pH range from 7.2 −8.1 indicating their physiological adaptability. *In situ* observations and onboard experiments on the entire phytoplankton community may not document the response of a particular species to a combination of stress factors. Hence, experimental studies on single species under controlled conditions are needed to improve our mechanistic understanding of their physiological plasticity and may help to envisage the community shift and niche occupancy in the future Arctic. Because, experimental studies on *C. gelidus* investigating the growth dynamics and carbon capture potential under variable light and CO_2_ levels inside controlled laboratory conditions are rare to fill this lacuna, the present experiment was conducted.

Earlier studies on Arctic phytoplankton reveal that experimental setups largely differed in terms of selecting growth conditions like light (continuous or a definite light-dark cycle), temperature (0−8° C), and type (on-deck incubation, or laboratory condition) either in monoculture or natural population. And despite such a wide range of variability in growth conditions, growth responses were comparable under variable CO_2_ supply (Hoppe et al., 2018b and references therein). Thus, different experimental conditions may not be a major factor affecting species-specific responses to ocean acidification in the Arctic phytoplankton. In this study, the growth response (elemental composition, pigment signature, and photophysiology) of *C. gelidus* were planned to be monitored under variable irradiance (50 and 100 µmol photons m^-2^ s^-1^) and *p*CO_2_ (240 and 900 µatm) levels over 12 days at 2°C temperature under 10:14 hrs light-dark cycle in the laboratory condition. The 12-day growth phase was initially nutrient enriched and the last few days were with depleted nitrogen resources which may not be persistent in the future Arctic except at the end of a phytoplankton bloom. Monitoring this phase was planned to understand 1) how this bloom-forming diatom maintains per cell carbon accumulation and 2) if there was any indication of dissolved organic carbon (DOC) release under such conditions.

## Materials and methods

### Experimental setup

**Figure 1:**
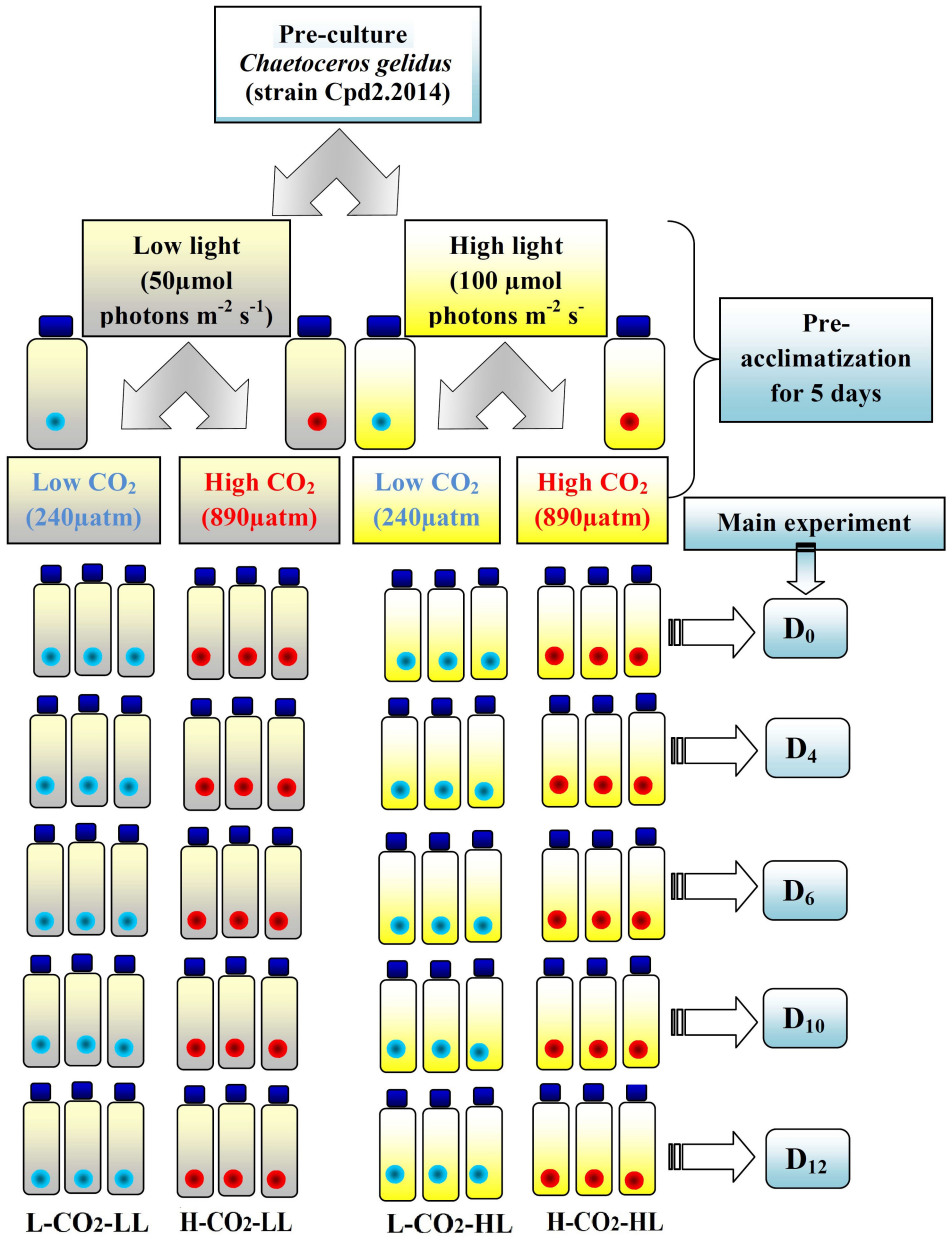
Schematic representation of the experimental pattern showing the replicates (3) and variables for each treatment. The blue and red marked bottles represent the low CO_2_ (L-CO_2_) and high CO_2_ (H-CO_2_) levels, respectively. The light and bright yellow shades indicate the low light (LL) and high light (HL) levels. The right side blue shaded boxes show the day of sampling.

The present study was conducted at the Biological Oceanography Division, Alfred Wegener Institute of Polar and Marine Research, Bremerhaven, Germany between July –Aug 2015. A chain-forming centric diatom *C. gelidus* (earlier known *C. socialis*) strain Cpd2.2014 isolated from the Kongsfjord, Svalbard in 2014, was used for this investigation. Almost 100 L of aged Arctic water (collected from Kongsfjord, Svalbard in 2014, stored at 4°C in dark) was filtered using a 0.2 µm filter connected to a peristaltic pump into the acid-cleaned 20 L carboys and were supplemented with nutrients and trace metal mix according to the F/2 medium. Pre-cleaned Borosilicate 1 L bottles (60 No.) were filled with this water with the help of a clean Tygon tube and kept tightly closed (without headspace) to set “2 light levels × 2 CO_2_ levels” (in triplicate) (**Figure 1**). A CO_2_ pipeline with 1000 µatm *p*CO_2_ was used with multi-channels to each bottle with a particular bottle top glass apparatus. pH values (NBS scale) were monitored in each bottle using a pH meter connected to a combined electrode and were calibrated using the standard buffer solutions (NBS). Within overnight, the pH values dropped from their initial value of 8.22 to 7.76 ± 0.004. The details of the carbon chemistry parameters are given in **Table 1**. The *p*CO_2_ level in the ambient water was calculated as 238 µatm and will be regarded as ≈ 240 µatm (L-CO_2_); in the high CO_2_ treatment it was 890 µatm and will be regarded as ≈ 900 µatm (H-CO_2_).

**Table 1:** Initial and final carbon chemistry parameters (n =3-10) calculated using CO2SYS (Pierrot et al., 2006) over the experimental period (considering salinity 31.62 ± 0.08; dissolved silicate and phosphate levels were 85 µM ± 3.7, 4.50 ± 0.08 µM, respectively, average temperature 2.07 ± 1.3 (n=2684) was recorded over the experimental period).

	*p*CO_2_ (µatm)		Dissolved inorganic carbon DIC (µmol kg^-1^)		Total AlkalinityTA (µmol kg^-1^)		pH (NBS)		CO_2_ (µmol kg^-1^)	
Date	L-CO_2_	H-CO_2_	L-CO_2_	H-CO_2_	L-CO_2_	H-CO_2_	L-CO_2_	H-CO_2_	L-CO_2_	H-CO_2_
Light (50 µmol photons m^-2^s^-1^)
31July'15 (D_0_)	238±25	890±90	2171±19	2355±12	2382±19	2382±19	8.35±0.04	7.83±0.04	14.1±1.53	52.7±5.3
4 Aug'15 (D_4_)	184±10	746±71	2125±11	2335±11	2382±19	2382±19	8.44±0.02	7.90±0.03	10.9±0.6	44±4.2
6 Aug'15 (D_6_)	176±18	475±46	2107±31	2278±4.5	2372±14	2381±17	8.46±0.03	8.08±0.04	10.4±1	28±2.7
10 Aug'15 (D_10_)	90±36	382±42	1972±76	2274±18	2396±16	2412±5.7	8.71±0.13	8.17±0.04	5.34±2	22.6±2.5
12 Aug'15 (D_12_)	35±3.2	217±67	1772±6.3	2173±76	2410±24.4	2411±33	8.99±0.03	8.40±0.12	2.1±0.19	12.8±3.9
Light (100 µmol photons m^-2^s^-1^)										
31July'15 (D_0_)	238±25	890±90	2171±19	2355±12	2382±19	2382±19	8.35±0.04	7.83±0.04	14.1±1.53	52.7±5.3
4 Aug'15 (D_4_)	169±6.5	703±103	2109±7.5	2327±18	2382±19	2382±19	8.47±0.01	7.92±0.06	10±0.38	41±6
6 Aug'15 (D_6_)	164±22	509±100	2093±25	2283±17	2374±5.5	2380±12	8.48±0.05	8.06±0.06	9.75±1.3	30±5.9
10 Aug'15 (D_10_)	83±20	293±62	1975±61	2224±31	2413±7	2400±11	8.73±0.08	8.28±0.08	4.94±1.2	17.3±3.7
12 Aug'15 (D_12_)	39.7±2.3	167±54	1786±9.6	2077±25	2395±10	2392±7.97	8.96±0.01	8.49±0.11	2.35±0.14	9.92±3.2

*C. gelidus*, strain Cpd2.2014 culture was pre-acclimatized at both L-CO_2_ and H-CO_2_ levels under two light levels (50 µmol photons m^-2^s^-1^ and 100 µmol photons m^-2^s^-1^, white fluorescence light sources measured daily by a Li-COR sensor) for 5 days (> 5 generations at 2 °C (using temperature logger EBI-20-T) inside a temperature-controlled room. A similar protocol was also followed by Wolf et al. (2019). The light levels chosen for this study were within the moderate range and neither growth limiting nor inhibiting. Selecting two extreme light levels may lead to dissimilar growth dynamics and may not be comparable for such an experimental design. The cells were exposed to a 10 hrs light:14 hrs dark cycle. These light and temperature levels resemble the condition in end Aug to September in Svalbard. Wolf et al. (2019) considered 2°C as the present-day temperature. These pre-acclimatized cells were used for the main experiment which continued for 12 days (**Figure 1**). There were a total of four experimental sets with triplicate for each sampling day. Sampling was done on day zero (D_0_), day four (D_4_), day six (D_6_), day ten (D_10_), and day twelve (D_12_) without exposing the cells to any change in temperature and light levels. Individual bottles were completely used for everyday sampling. It was a closed batch culture experiment and the initial *p*CO_2_ level was set on the initial day. Dilute culture (<200 cell mL^-1^) was allowed to grow until nutrients and CO_2_ levels were exhausted.

### Analytical method

#### Carbon chemistry and dissolved inorganic nutrients

Total alkalinity (TA) values were measured in replicates using an Autotitrator (a titration Center with Titro Line alpha 05 plus, Autosampler TW alpha plus, and Software Titrisoft 2.71) from Schott Instrument GmbH, Germany by potentiometric titration (duplicate analysis) against the certified reference material (Dickson). Dissolved inorganic carbon (DIC) contents were measured using a coulometric acidification module (QuAAtro39 Continuous Segmented Flow Analyzer, SEAL Analytical, Germany) against a certified reference material (Dickson, Scripps Institute of Oceanography, USA). TA and DIC values were used to calculate the *p*CO_2_ and pH using the program CO2SYS (Pierrot et al., 2006) considering the levels of dissolved silicate and phosphate, salinity, and temperature. Dissolved inorganic nutrients were measured following the standard colorimetric methods using a Continuous Segmented Flow Analyzer with TP-2020 Plus, Autosampler, and AACE 7.09 Software from Seal Analytical, Germany against the certified reference materials.

#### Elemental analysis

For estimating particulate organic carbon (POC) and nitrogen (PON), 50-100 mL of sample water was filtered using a pre-combusted GF/F filter (at 500°C overnight) (25 mm) and stored in a clean Petri dish under -20°C till analysis. Before analysis, the filters were oven-dried at 40°C overnight and analyzed using an elemental analyzer (Euro EA, HEKAtech GmbH, Germany) following the method of Sharp (1974). The POC and PON contents were normalized to their respective filtration volumes. The ratio of C:N (µmol: µmol) was calculated POC and PON contents expressed in µmol L^-1^.

The POC-based specific growth rate (*µ* d^-1^) was calculated using the following equation


 (1) $$\left(\mu_{POC}d^{-1}\right)=\left[\ln\left(Final\;POC\;contents\right)\;-\ln\left(Initial\;POC\;contents\right)\right]\text{/time}\left(d\right)$$

#### Pigment analysis by high performance liquid chromatography

For pigment analyses, 200-500 mL sample water was filtered using GF/F filters and kept folded in quarters inside a cryo-vial and stored at -80°C till analysis. Pigment samples were extracted in 1.5 mL 99.9% HPLC grade acetone (Merck, Germany) overnight at -20°C. A small aliquot (50 µL) of the synthetic pigment canthaxanthin (95%, HPLC grade, Sigma) was added to each sample (after analysis Canthaxanthin quantity was used to normalize the pigment contents to their extraction volume). To this mixture, a small portion of silica beads (0.5 mm diameter from BioSpec Products, USA) was added and a tissue homogenizer (Precellys 24, France) was used to homogenate (5500 rpm for 20 s) the samples. The samples were cold centrifuged at -10°C for 15 min at 15000 rpm (Eppendorf, Germany). The supernatant was filtered using a 0.2 µm PTFE syringe filter to remove any cell debris and collected in an Eppendorf tube wrapped with aluminum foil to avoid photodegradation. Ammonium acetate buffer (1M) was mixed with an aliquot of sample extract (170 µL) in 1:1 (v/v) ratio before analysis. An HPLC model WATERS 600 (Waters Corporation, USA) fitted with a temperature-controlled autosampler (Waters 717 plus), a photo-diode-Array- Detector (Waters 2998), a fluorescence detector (Waters 2475), and a binary pump (Waters 1525) was used to detect the extracted pigments. A C8 column (4.6 × 100 mm) VARIAN Microsorb-MV 3 from Agilent Technologies, (Inc., Santa Clara, CA, USA) was used for reverse phase HPLC using solvent 1 (a mixture of 70% methanol and 30% ammonium acetate (1 M)) and solvent 2 (100% methanol) from Merck (HPLC grade, Germany). Individual pigments were identified against the retention time of the standard procured from DHI Water and Environment (Netherlands) and the corresponding area was used to calculate the concentration of individual pigment.

#### Dissolved organic carbon and nitrogen

Samples for dissolved organic carbon (DOC) were collected using a specialized filtration unit which was placed inside a glass cover to avoid contamination. Pre-combusted GF/F filters were used to filter the particulate fraction and collected in a pre-acid cleaned hard plastic bottle at -20°C till analysis. DOC and total dissolved nitrogen (TDN) in the filtrate were determined by high-temperature catalytic oxidation (HTCO) and subsequent non-dispersive infrared spectroscopy and chemiluminescence detection using a Shimadzu TOC-VCPN analyzer fitted with an autosampler. To remove inorganic carbon the samples (6.5 mL) were acidified with HCl and sparged with oxygen for 5 min within the autosampler. 50 µL sample volume was injected directly into the catalyst (heated to 680°C). Final DOC concentrations were average values of triplicate measurements. If the standard variation or the coefficient of variation exceeded 0.1 µM or 1%, respectively, up to 2 additional analyses were performed and outliers were eliminated. After each batch of six samples one DSR (Deep Sea Water Reference Material, Hansell Research Lab, University of Miami, US), one Milli-Q blank, and one potassium hydrogen phthalate standard were measured. The limit of quantification (LOQ) was 7 µM for DOC and 11 µM for TDN and the accuracy was ±5%.

#### The maximum quantum yield of photosystem II (Fv/Fm)

The photophysiological responses in terms of photosynthetic quantum yield (F_v_/Fm) of the diatom culture were measured using a Xenon-PAM fluorometer (WALZ GmbH, Germany) fitted with a magnetic stirrer and temperature control unit. The samples were dark-adapted for 10 min before fluorescence response was measured. The maximum quantum (F_v_/F_m_) yield was calculated as = (F_m_ – F_0_)/F_m_ where F_m_ and F_0_ represent the maximum and minimum fluorescence (Krause and Weis, 1991).

#### Cell density

Samples for cell density measurements (50 mL) were collected and preserved with Lugol’s iodine (4%) and kept in a refrigerator at 4°C. Cells were counted using an “Utermohl counter (3 mL, Hydro-Bios, Kiel, Germany) under an inverted microscope (Carl Zeiss, Germany) under ×400 magnification. The specific growth rate (*µ* d^-1^) was calculated using the following equation:


 (2) $$\left(\mu_{cell\;no.}d^{-1}\right)=\left[\ln\left(Final\;cell\;density\right)\;-\ln\left(Initial\;cell\;density\right)\right]\text{/time}\left(d\right)$$

### Statistical analysis

The individual data set was checked for normality and variance using the Shapiro-Wilk normality test and F-test, respectively. In case the data sets were normally distributed (null hypothesis accepted), showing equal variance, a two-way Analysis of Variance (ANOVA) with replication was performed for testing the level of significance due to light and CO_2_ levels keeping the *p*-values at 0.05 (95% confidence level). Those parameters did not pass through the former check and revealed a non-normal distribution pattern (rejecting the null hypothesis) of distribution, the nonparametric Two-sample Mann-Whitney-U test was performed to understand if the observed differences were statistically significant (*p*-values at 0.05; at 95% confidence level).

## Results

### Growth response

**Figure 2:**
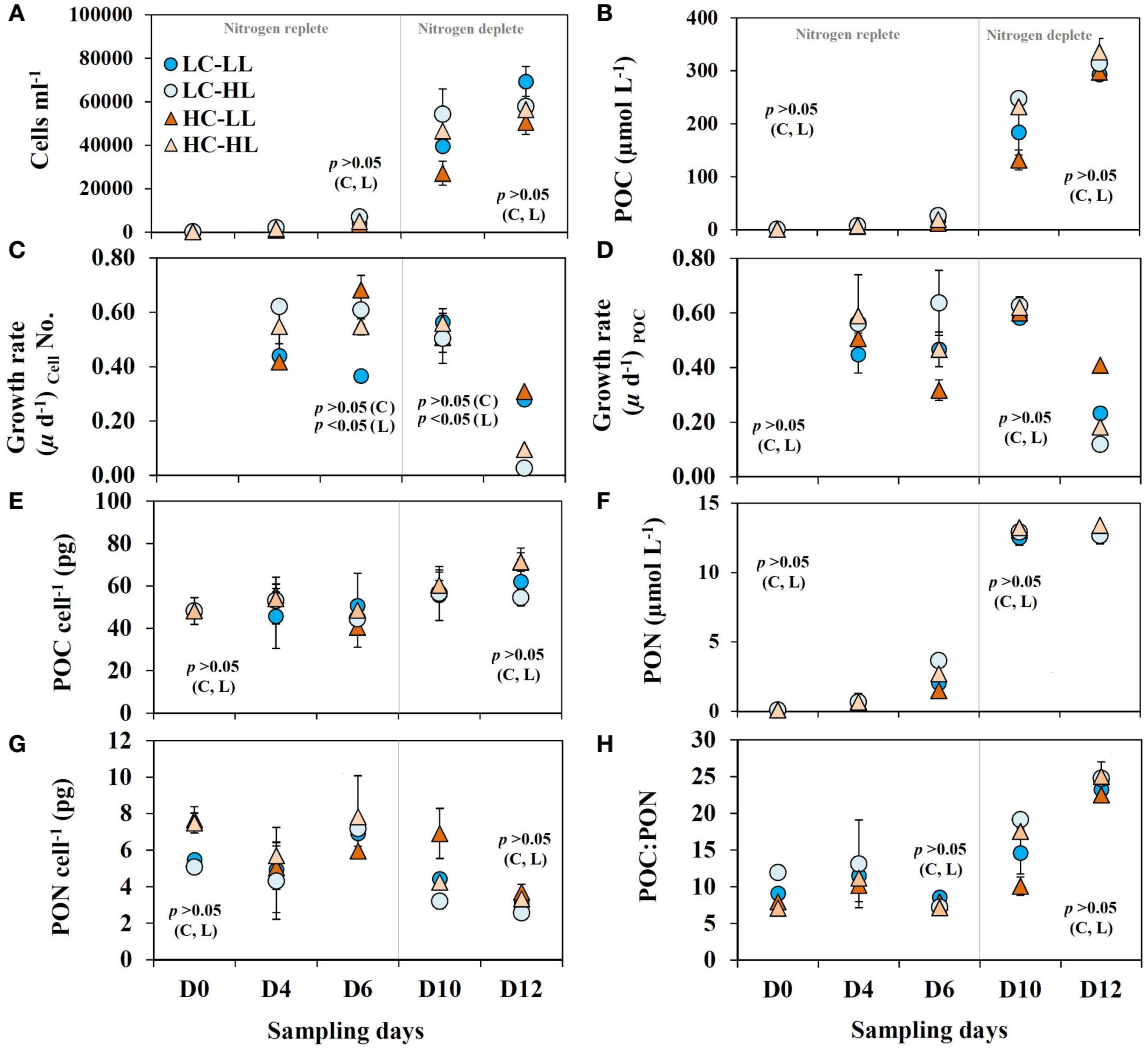
Growth dynamics of *C*. *gelidus* over the experimental period (total 12 days, D_0_ –D^12^) under two different light conditions [low light (LL) = 50 µmol m^-2^ s^-1^, high light (HL) =100 µmol m^-2^ s^-1^] and CO_2_ levels [low CO_2_ (LC) ≈240 µatm and high CO_2_ (HC) ≈900 µatm levels]. **(A)** cell density; **(B)** particulate organic carbon (POC) contents; growth rates calculated based on **(C)** cell number and **(D)** POC contents; **(E)** POC contents per cell; **(F)** particulate organic nitrogen (PON); **(G)** PON contents per cell; **(H)** the ratio between POC and PON (C:N) (n =3; ± SD). The level of significance (*p*-values) related to CO_2_ and light levels are shown as C and L, respectively. The gray line demarcates the nitrogen replete and deplete phases.

Like other diluted batch culture experiments, the present experiment also started with a very low cell density (<200 cell mL^-1^) which increased significantly (approximate 60,000 cell mL^-1^) in all treatments over the incubation period (**Figure 2A**). A clear increase in pH (**Table 1**) was noticed from D_0_ to D_12_ with concomitant enhancement in cell density, POC and pigment contents in all treatments. The experiment started with POC concentrations<1 µmol L^-1^ and within 12 days of the experimental period more than 300 µmol L^-1^ POC production was recorded (**Figure 2B**). There was no statistically significant difference (*p*>0.05) in POC accumulation rates from the different light and CO_2_ levels during the growth phase. Cell density (**Figure 2A**) and POC concentrations (**Figure 2B**) showed a typical exponential growth pattern without any significant difference within the treatments (*p*>0.05) suggesting that increasing CO_2_ supply or enhanced light did not impact the cell division rates and carbon biomass accumulation in this diatom. The corresponding growth rates based on the cell density (**Figure 2C**) and POC contents (**Figure 2D**) increased exponentially in all treatments and revealed high linearity (*p*<0.05). This observation suggests that overall growth performance was almost comparable under variable light and CO_2_ levels and the rate of cell division was coupled with photosynthetic and POC accumulation rates. The highest growth rates were noticed till D_6_ and then there was a significant drop noticed in all treatments. Per cell POC content (**Figure 2E**) showed the highest values (61 ± 6 pg POC cell^-1^) in the last two sampling days and this value was more than 26% higher (*p*<0.05) than the values (48.8 ± 4 pg POC cell^-1^) noted during the first three sampling days.

PON contents showed an exponential increase (**Figure 2F**) from an initial value of< 0.1 µmol L^-1^ up to 13 µmolL^-1^. These increasing trends were steady up to D_10_ in all treatments and did not show any further enhancement. The concentrations of PON per cell (**Figure 2G**) within D_4_ to D_10_ were comparable and showed an average value of 6 ± 1.2 pg PON cell^-1^ and this value was reduced on D_12_ in all treatments. The average POC: PON values (**Figure 2G**) within the first three sampling days (9.2 ± 2) were slightly higher than the classical Redfield ratio of 6.63 and got almost doubled in the last two sampling days (19.6 ± 5). There was no statistically significant correlation (*p*>0.05) found between POC: PON values and light or CO_2_ levels.

### Photophysiological response

#### Photosynthetic quantum yield (Fv/Fm)

**Figure 3:**
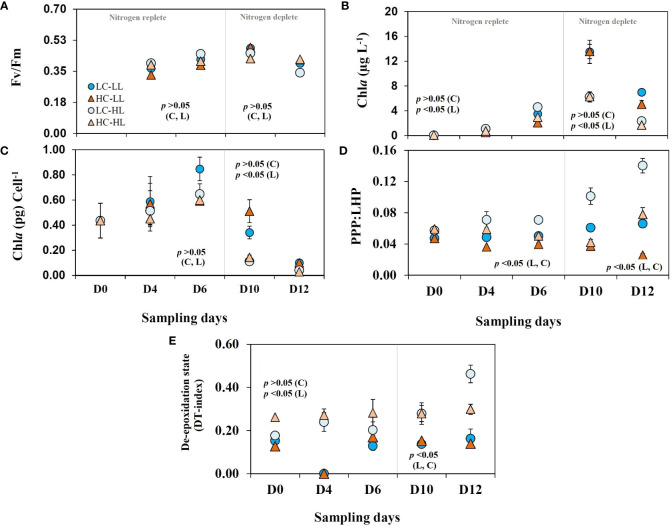
Photophysiogy and pigment signature of *C*. *gelidus* over the experimental period under two different light conditions [low light (LL) = 50 µmol m^-2^ s^-1^, high light (HL) =100 µmol m^-2^ s^-1^] and CO_2_ levels [low CO_2_ (LC) ≈240 µatm and high CO_2_ (HC) ≈900 µatm levels]. **(A)** Fv/Fm values; **(B)** Chl*a* contents; **(C)** Chl*a* contents per cell; **(D)** the ratio between photoprotective and light harvesting pigment contents (PPP : LHP) and **(E)** diatoxanthin index [DT/(DD+DT)] (n =3; ± SD). The level of significance (*p*-values) related to CO_2_ and light levels are shown as C and L, respectively. The gray line demarcates the nitrogen replete and deplete phases.

FigureFv/Fm values in all treatments (**Figure 3A**) showed a gradual increase from an initial value of 0.37, and the highest values (0.48) were seen on D_10_ followed by a decrease on D_12_ (0.4) suggesting the end of the growth phase. There was no statistically significant difference found between the treatments.

### Pigment signature

Variable light levels exerted stronger impacts (*p*<0.05) on Chl*a* concentrations than CO_2_ (*p*>0.05). The concentrations of Chl*a* (**Figure 3B**) increased from an initial value of< 0.2 µg L^-1^ up to 15.7 µg L^-1^ over the incubation period. The average Chl*a* concentration up to D_6_ was 1.78 ± 1.5 µg L^-1^ and a difference (*p*<0.05) was noticed on D_10_ when the average concentrations of Chl*a* were 13.5 ± 1.4 µgL^-1^and 6.27 ± 0.63 µgL^-1^ at L-CO_2_ and H-CO_2_ treatments, respectively. On D_12,_ there was a decrease in Chl*a* values in all treatments. The Chl*a* content (3.74 ± 0.6 µgL^-1^) in HL incubated cells (considering both CO_2_ levels) was ≈ 38% lower than that of LL treatment (*p*<0.05). Per cell Chl*a* contents (**Figure 3C**) was 0.44 pg on D_0_ and increased linearly up to D_6_ and decreased steadily thereafter in all treatments with variability. From D_0_ to D_6_ per cell Chl*a* values did not reveal any difference between the treatments (*p*>0.05); however, the values from D_10_ and D_12_ were significantly different (*p*<0.05). The LL incubated cells from both CO_2_ levels showed almost three times higher Chl*a* (0.26 ± 0.19 pg cell^-1^) contents than that of HL treated cells (0.082 ± 0.05 pg cell^-1^) without any significant impact of variable CO_2_ levels (*p >*0.05).

### Accessory pigments

The accessory pigments of the diatom were fucoxanthin (Fuco), chlorophyll*c*_2_ (Chl*c*_2_), diadinoxanthin (DD), diatoxanthin (DT), and *β*-carotene. Among these pigments, the first two are light-harvesting pigments (LHP), and the last three work as photoprotective pigments (PPP). All these pigments showed significant variation in response to light and CO_2_ levels. The ratios of PPP : LHP (**Figure 3D**) were impacted by both light and CO_2_ levels (*p*<0.05). The highest value of PPP : LHP ratio was noticed in the cells grown under L-CO_2_-HL treatment (average 0.093) and the minimum values (average 0.036) were seen in the cells incubated at H-CO_2_-LL treatments. The H-CO_2_ incubated cells showed higher LHP accumulation relative to PPP resulting in a lower PPP : LHP ratio. The values of de-epoxidation states [DT/(DT+DD)] (**Figure 3E**) were almost half (*p*<0.05) under the LL treated cells (0.15 ± 0.02) than that of HL (0.33 ± 0.09) and CO_2_ levels did not show significant impact (*p >*0.05) on this ratio.

### TDN, DOC, and DON

**Figure 4:**
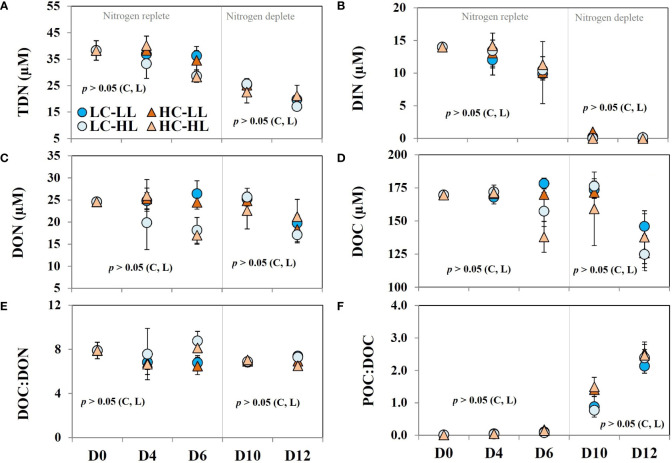
**(A)** total dissolved nitrogen (TDN) contents; **(B)** dissolved inorganic nitrogen (DIN) contents; **(C)** dissolved organic nitrogen (DON); **(D)** dissolved organic carbon contents (DOC); **(E)** the ratio of DOC : DON; **(F)** the ratio of POC : DOC over the experimental period under two different light conditions [low light (LL) = 50 µmol m^-2^ s^-1^, high light (HL) =100 µmol m^-2^ s^-1^] and CO_2_ levels [low CO_2_ (LC) ≈240 µatm and high CO_2_ (HC) ≈900 µatm levels] (n =3; ± SD). The level of significance (*p*-values) related to CO_2_ and light levels are shown as C and L, respectively. The gray line demarcates the nitrogen replete and deplete phases.

Total dissolved nitrogen (TDN) concentration was 38.3 ± 3.7 µM in the initial Arctic water and decreased steadily to half on D_12_ (**Figure 4A**). To derive the values of dissolved organic nitrogen (DON), the concentrations of DIN were subtracted from the TDN values. DIN (**Figure 4B**) contributed to almost 36% of TDN and the rest was DON (**Figure 4C**). The concentrations of DIN showed an exponential declining trend coupled with biomass buildup in all treatments without any significant difference. DIN became almost undetectable in most of the treatments on D_12_. The initial value of the DON was 24.6 ± 0.11 µM and no significant difference was seen under LL treatments (both CO_2_ levels) until D_10_. However, on D_12_ almost a 27% decrease in DON concentrations was noticed under both CO_2_ levels. In the case of HL-treated cells, the trend of DON values was different than noticed under LL conditions. In L-CO_2_-HL treatment, ≈a 20% decrease in DON contents was noticed on D_4_ and D_6_ but increased again to its initial value on D_10_ followed by a decline on the final day. Under H-CO_2_-HL treatment, a significant decrease in DON was noticed on D_6_ and the level increased again on D_10_ followed by a further decrease on D_12_. The average values of DON noticed on D_12_ in all treatments were significantly (*p*<0.05) lower (28%) than the initial values.

The initial DOC concentration (**Figure 4D**) in the Arctic water was nearly 170 ± 5 µM. On D_4_, DOC concentrations showed negligible variability (<1%) from the initial concentrations (*p*>0.05). Minor variability was seen under LL conditions on D_6_ from the concentrations noticed on D_4_. However, under HL, the decrease in DOC concentrations was >8% and ≈ 20% from the previous day. DOC values did not change beyond 5% on D_10_ in all treatments and there was no statistically significant variability among the treatments. On the last day (D_12_), DOC concentrations decreased to 30 – 40 µM in all treatments corresponding to a 27% decrease from the initial average but without any statistical significance based on light or CO_2_ levels. DON and DOC revealed a linear positive correlation (*p*<0.05) and the ratio of DON : DOC (**Figure 4E**) varied close to 7.28 on average.

The ratio of POC : DOC (**Figure 4F**) was extremely low (0.005 ± 0.0012) on the initial day. With the progression of the experiment, POC concentration increased with a corresponding enhancement in POC : DOC ratio, and an eight-fold enhancement was noticed on D_4_. A clear exponential increase was noticed in POC : DOC ratio and the maximum value (≈2.5) was seen on D_12_. The decrease in DOC on the last day with a concomitant increase in POC probably resulted in such a considerable increase in this ratio.

## Discussion

### Growth under nutrient-enriched phase

The overall growth response depicted that the Arctic diatom *C. gelidus* can grow efficiently at low as well as high pH/CO_2_ levels without compromising its carboxylation and cell division rates. Such observation indicates that the CO_2_ resilience of marine diatoms can be natural in some diatoms, and the strategy for resilience could be species-specific (Valenzuela et al., 2021). The photosynthetic performance as reflected in POC content per cell was comparable under both CO_2_ levels. Likewise, Hoppe et al. (2018b) documented a change in *p*CO_2_ level from 240 to 970 µatm yielded no significant difference in net primary production under nutrient-enriched conditions in an Arctic phytoplankton community. However, there was high species-specific as well as strain-specific diversity in the observed responses. Wolf et al. (2017) noticed that variable *p*CO_2_ (180 -1400 µatm) levels did not impact per cell POC production at 3°C in the Arctic diatom assemblage containing *C. gelidus* (mentioned as *C. socialis*). These observations hinted at the possibility of existing highly efficient mechanisms for acquiring and fixing sufficient CO_2_ even at a very low *p*CO_2_ level. As has been mentioned earlier that marine diatoms use efficient and diverse CCMs to accumulate higher CO_2_ levels over O_2_ close to Rubisco (Hopkinson et al., 2016; Young et al., 2016; reviewed by Matsuda et al., 2017), and similar mechanisms may also exist in this diatom.

Although CCMs components were not investigated in this study, the possibility of operating CCMs becomes obvious from the final POC value, particularly in the low CO_2_ treatment. The accumulated POC can be considered as the net organic carbon buildup preceded by carboxylation excluding respiratory loss and DOC leaching if any. Importantly, POC : DOC ratio indicated insignificant DOC leaching compared to POC production in this diatom under both CO_2_ levels. At the L-CO_2_ treatment, the final POC content (303 µmol L^-1^ or 296 µmol kg^-1^) was about 20 times higher than the initial CO_2_ concentration (14 µmol kg^-1^) and importantly, CO_2_ was not supplemented further. Considering the final CO_2_ concentration (≈ 2 µmol kg^-1^) from the L-CO_2_ treatment, almost 280 µmol of CO_2_ seemed to be acquired *via* CCMs mostly utilizing abundant bicarbonate ions. However, there could be some diffusive loss of CO_2_ too. This fact strengthened the possibility of operating CCMs in *C. gelidus*. During a spring diatom bloom in the Western Antarctic Peninsula, it was observed that when CO_2_ concentration was less than 6 µM, CCMs could supply CO_2_ almost at the Rubisco saturation level (Kranz et al., 2015). Similarly, in the present study, *C. gelidus* was likely to perform a steady carbon fixation rate under the *p*CO_2_ level that was much lower than the atmospheric level, particularly after D_6_ when the CO_2_ level dropped to<6 µmol kg^-1^. Nevertheless, the absence of any CO_2_ fertilization impact on growth parameters indicated that the diatom species did not benefit from increased CO_2_ supply. As has been hypothesized (Hopkinson et al., 2011 and references therein) that a higher diffusive CO_2_ influx could benefit cells by increasing CO_2_ supply, decreasing diffusive CO_2_ loss, and downregulating the functioning of CCMs to save the energetic cost and collectively may increase carboxylation and growth rates. However, it has been observed that not all phytoplankton show such growth impacts in response to enhanced CO_2_ supply (Tortell et al., 2008; Hoppe et al., 2018a). Likewise, the study by Tortell et al. (2008) noted that the DIC uptake was not correlated with *in situ p*CO_2_ variabilities in a phytoplankton community from the Ross Sea (Southern Ocean). In the same study, the authors observed no impact of an eight-fold increment in *p*CO_2_ supply on the bicarbonate uptake rate. Such observation indicates that DIC acquisition *via* CCMs may not be altered for all phytoplankton at the elevated CO_2_ supply and even if CCMs downregulation occurs, that may not change polar diatom growth rates (Young et al., 2015).

Other than acquiring CO_2_ *via* CCMs, the type of Rubisco present in phytoplankton is also responsible to a large extent for fixing CO_2_ efficiently. Diatoms possess one of the most proficient forms of Rubisco (red algal type ID) (Young et al., 2016) and it was found that Rubisco in some Arctic diatoms including *C. gelidus* possesses a high specificity factor for CO_2_ relative to O_2_ (Valegård et al., 2018).

### Growth under nitrogen limitation

The growth phase was mostly declined by nitrogen depletion. The observed growth response during this period when the *p*CO_2_ levels were already low in all treatments, may not be comparable to nitrogen limitation under the ocean acidification scenario (**Table 1**). And this phase was monitored, particularly to know if POC : DOC fractionation was impacted in *C. gelidus*. Under nitrogen limitation when synthesizing nitrogenous macromolecules like proteins become constrained, marine diatoms may release a high amount of DOC relative to POC (Myklestad, 2000). In this study, due to the presence of natural DOC in the Arctic water, the initial DOC level was higher than POC contents (low POC : DOC ratio). With the progression of the growth phase POC : DOC ratio increased significantly suggesting insignificant DOC leaching throughout the growth phase. Likely, *C. gelidus* was capable of converting fixed carbon to storage macromolecules under depleted nitrogen levels.

A simple mass balance calculation based on the classical Redfield ratio shows that under ideal conditions, 16 µmol of DIN is needed to accumulate 106 µmol of POC. Likewise, to produce 300 µmol POC (as seen at the end of the growth phase) ≈ 45 µmol of DIN shall be needed. But the amount of DIN was one-third of it and was consumed within six days of cultivation. PON contents per cell increased from D_0_ to D_6_ coupled with depleted DIN levels suggesting a steady nitrogen uptake during the exponential growth phase. However, it is likely that after D_6_ the growth of *C. gelidus* was mostly nitrogen limited, yet POC content per cell did not decrease suggesting that carbon fixation and storage were unaffected. Similarly, Li et al. (2012) reported in a model diatom that under low and high CO_2_ levels with sufficient nitrogen supply, per cell POC and PON contents did not show any significant change. However, the authors noticed a considerable decrease in per-cell nitrogen content under similar treatments when nitrogen supply was insufficient and thus supports the present study. Diatoms possess several strategies to overcome nitrogen starvation (Smith et al., 2019) and the cellular amino acid pool may be recycled to provide a nitrogen source to the cell (Alipanah et al., 2015).

Diatoms use stored carbon usually as chrysolaminarin or lipids (Kroth et al., 2008) and under nitrogen starvation, they reprogram the storage carbon from nitrogenous compounds like proteins and nucleic acid to neutral lipids (Valenzuela et al., 2012). It was recorded in a model marine diatom that under nitrogen stress, the cell allocated more nitrogen towards nitrogen assimilation enzymes compared to the photosynthetic apparatus, and the freshly produced carbon is utilized to synthesize more lipids (Levitan et al., 2014). Consequently, POC would increase at the cost of PON leading to a higher POC : PON ratio and is in agreement with our observation, particularly after D_6_ (**Figure 2H**). Alipanah et al. (2015) also noticed a significant increase in POC : PON ratio in a model diatom under nitrogen exhaustion. Importantly, phytoplankton may also utilize Rubisco as a nitrogen reservoir under nitrogen stress and may recycle cellular organic nitrogen (Herrig and Falkowski, 1989). And high Rubisco contents (˜36%) as has been seen in Arctic diatoms (Gerecht et al., 2019) could be advantageous under nitrogen starvation. However, the existence of such nitrogen metabolism in *C. gelidus* is subjected to further investigation. Diatoms may also uptake organic nitrogen under nitrogen limitation (Cochlan et al., 2008) and also possess an animal-like urea cycle (Armbrust et al., 2004; Allen et al., 2011). Schiffrine et al. (2020) noticed that *C. gelidus* (strain RCC2046, Beaufort Sea isolate) was able to grow only at urea in the absence of other nitrogen sources. On D_12_ the decline in DON contents under undetectable DIN content indicated probable DON consumption to supplement inorganic nitrogen. Likewise, it was observed in the Arctic that, DON may support nearly 60% of the primary production in the top 10 m of the water column (Le Fouest et al., 2013).

### Photophysiological response

Arctic phytoplankton, particularly diatoms (Lacour et al., 2019) are highly efficient in adjusting to fluctuating light and are capable of keeping their photosynthetic apparatus functional over the dark winter period (Hoppe, 2021). In the present study, the comparable Fv/Fm values under variable light and CO_2_ levels suggested that *C. gelidus* efficiently maintained photophysiological performances. Croteau et al. (2022) conducted a study on five ecologically important diatoms from the Arctic waters and revealed that *C. gelidus* showed necessary photoadaptable strategies to bloom in the subsurface chlorophyll maxima. A drop in Fv/Fm values on D_12_ can be attributed to nitrogen limitation that may potentially impact photosystem II functioning (Falkowski et al., 2017).

Marine diatoms respond quickly to fluctuating light *via* short-term photoacclimation processes without compromising their photosynthetic performance (Raven and Geider, 2003). Diatoms predominantly use a xanthophyll cycle containing DD. Under light stress, the de-epoxidation of DD to DT is initiated by the pH gradient of the thylakoid (Lavaud et al., 2002) to dissipate excessively absorbed light energy as heat in a non-radiative process (non-photochemical quenching; NPQ) which is usually indicated by the occurrence of DT (Lavaud et al., 2007; Niyogi and Truong, 2013). In the present experiment, DT production under HL was significantly higher than observed in the LL-treated cells suggesting the activation of NPQ. Croteau et al. (2020) reported that *C. gelidus* exhibited higher DT production and NPQ when grown under 50 µmol photons m^-2^ s^-1^ compared to that of 15 µmol photons m^-2^ s^-1^ at close to freezing temperature. Similar results were also noticed in other bloom-forming Arctic diatoms (Lacour et al., 2018; Lacour et al., 2019). Unaffected growth under variable light and CO_2_ levels were also reported in other marine diatoms (Ihnken et al., 2011; Valenzuela et al., 2018) including *C. gelidus* from the Arctic (Hoppe et al., 2018b). Kvernvik et al. (2020) and Wolf et al. (2022) showed that the open water diatoms grew better under the combined stress of light and CO_2_ compared to sea ice diatoms in the Arctic. Contrarily, a few studies reported that variable light intensity-induced NPQ can be modulated by CO_2_ levels in some marine diatoms (Hoppe et al., 2015 and references therein).

The occurrences of the minimum Chl*a* contents in the HL-treated cells irrespective of CO_2_ levels suggested that light-harvesting was downregulated in response to enhanced photons flux density from 50 to 100 µmol m^-2^ s^-1^ (Geider et al., 1996; Falkowski and Chen, 2003; Brunet et al., 2011). Likewise, a significant decrease in per cell Chl*a* content was noticed in a marine diatom after eight-fold increased light intensity from 50 µmol m^-2^s^-1^ (Li and Campbell, 2013). Contrarily, when photons flux density is below growth saturating levels, Arctic diatoms increase light harvesting pigment contents and downregulate photoprotective pigment synthesis (Lacour et al., 2019) as noticed in the present study. The bulk and per cell Chl*a* contents increased linearly up to D_6_ (nitrogen replete phase) and these trends reversed with depleting nitrogen levels. Chl*o*rophyll synthesis is directly dependent on nitrogen availability (Geider et al., 1997) and can be downregulated while upregulating the synthesis of photoprotective carotenoids under nitrogen stress. Under the combined stress of nitrogen limitation and light marine diatom upregulate photoprotection related genes (Alipanah et al., 2015). Decreased per cell Chl*a* and photosynthetic rates under nitrogen starvation to adjust to elevated CO_2_ levels were recorded in a model marine diatom (Hennon et al. (2014). In *C. gelidus* likely that photosynthesis remained steady as per cell POC contents remained unaffected in response to elevated CO_2_ levels, changing irradiance, and depleted nitrogen resources. Because of such fitness to climate change variables, *C. gelidus* could be a potential bloom-forming species in the future Arctic water.

## Conclusions

In the Arctic, besides increasing temperature, light, and CO_2_ levels are the most rapidly varying climate change variables that control phytoplankton growth. Specifically, the accelerated rate of ocean acidification is an alarming issue. Thus, phytoplankton response study to multiple stressors is an important issue to investigate. Any species showing resilience to these factors may be successful in growing in future Arctic waters. We show that the bloom-forming Arctic diatom *C. gelidus* is resilient to changing CO_2_ and light levels and are consistent with earlier reports. The high light level chosen for this experiment was not at the inhibitory level, and variable light alone and in combination with increasing CO_2_ levels did not alter photosynthetic performances. The overall results also depict that the photosynthetically fixed carbons were subsequently converted to biomass as POC contents increased and there was no indication of enhanced DOC leaching. Comparable POC contents per cell under the variable CO_2_ levels suggested that *C. gelidus* did not benefit from increasing CO_2_ levels. The signal of DON consumption under nitrogen starvation is an important observation and needs to be investigated further. Such a high level of physiological plasticity in carbon and nitrogen metabolism can make *C. gelidus* an “ecologically resilient” species in the future Arctic water. However, further studies at the molecular level on this species are highly recommended to improve our mechanistic understanding of its carbon and nitrogen metabolism under multiple stressors.

## Data availability statement

The original contributions presented in the study are included in the article/supplementary material. Further inquiries can be directed to the corresponding author.

## Author contributions

The author has written the research proposal, secured the funding and conducted the experiment including the major analysis. The data interpretation and manuscript preparation was also done by the author. The other academic and technical help from the host laboratory has been acknowledged.

## Acknowledgment

This study was funded by Deutscher Akademischer Austauschdienst (DAAD) or German Government Exchange service under the re-invitation program, 2015 for former long-term fellowship holders. The laboratory facilities and other technical support were provided by the Biological Oceanography Division, Alfred Wegener Institute of Polar and Marine Research (AWI). The active support provided by Professor Anya Waite to get this funding and also to conduct the entire work at AWI is thankfully acknowledged. I am particularly thankful to Mrs. Susanne Spahic for her tireless effort to execute this experiment. I also acknowledge the kind support provided by Dr. Boris Koch (Ecological Chemistry Division, AWI) for analyzing the dissolved organic carbon and nitrogen and also for his scientific advice regarding data interpretation. All technical support and scientific advice for experimental planning extended by Drs. Bjorn Rost and Sebastian Rokitta are gratefully acknowledged. The active support from Mrs. Tanja Glawatty to complete this endeavor is highly appreciated. The technical assistance provided by Ms. Claudia Burau, Ms. Christiane, and Ms. Sonja is thankfully acknowledged. I am also thankful to the director of CSIR-NIO for his encouragement and support. The NIO contribution number is 6986. The valuable comments and suggestions by the anonymous reviewers are gratefully acknowledged.

## Conflict of interest

The author declares that the research was conducted in the absence of any commercial or financial relationships that could be construed as a potential conflict of interest.

## Publisher’s note

All claims expressed in this article are solely those of the authors and do not necessarily represent those of their affiliated organizations, or those of the publisher, the editors and the reviewers. Any product that may be evaluated in this article, or claim that may be made by its manufacturer, is not guaranteed or endorsed by the publisher.
